# Reparative properties of human glioblastoma cells after single exposure to a wide range of X-ray doses

**DOI:** 10.3389/fonc.2022.912741

**Published:** 2022-08-04

**Authors:** Galina Pavlova, Alexandra Belyashova, Ekaterina Savchenko, Dmitri Panteleev, Dzhirgala Shamadykova, Anna Nikolaeva, Svetlana Pavlova, Alexander Revishchin, Denis Golbin, Alexander Potapov, Natalia Antipina, Andrey Golanov

**Affiliations:** ^1^ Nikolay Nilovich (N.N.) Burdenko National Medical Research Center of Neurosurgery (NMRCN), Moscow, Russia; ^2^ Laboratory of Neurogenetics and Genetics Development, Institute of Higher Nervous Activity and Neurophysiology of Russian Academy of Sciences, Moscow, Russia; ^3^ Department of Medical Genetics, Sechenov First Moscow State Medical University, Moscow, Russia

**Keywords:** human glioblastoma cell cultures, radiation therapy, Rad51, Ku70, Ku80

## Abstract

Radiation therapy induces double-stranded DNA breaks in tumor cells, which leads to their death. A fraction of glioblastoma cells repair such breaks and reinitiate tumor growth. It was necessary to identify the relationship between high radiation doses and the proliferative activity of glioblastoma cells, and to evaluate the contribution of DNA repair pathways, homologous recombination (HR), and nonhomologous end joining (NHEJ) to tumor-cell recovery. We demonstrated that the GO1 culture derived from glioblastoma cells from Patient G, who had previously been irradiated, proved to be less sensitive to radiation than the Sus\fP2 glioblastoma culture was from Patient S, who had not been exposed to radiation before. GO1 cell proliferation decreased with radiation dose, and MTT decreased to 35% after a single exposure to 125 Gγ. The proliferative potential of glioblastoma culture Sus\fP2 decreased to 35% after exposure to 5 Gγ. At low radiation doses, cell proliferation and the expression of RAD51 were decreased; at high doses, cell proliferation was correlated with Ku70 protein expression. Therefore, HR and NHEJ are involved in DNA break repair after exposure to different radiation doses. Low doses induce HR, while higher doses induce the faster but less accurate NHEJ pathway of double-stranded DNA break repair.

## Introduction

### Glioblastoma

Glioblastoma is one of the most common primary tumors of the brain with poor prognosis. The yearly incidence rate of glioblastoma is about 3.2 per 100,000 persons (1). More radical surgery and aggressive chemo- and radiation therapy insignificantly improve prognosis in terms of overall and disease-free survival. In the great majority of cases (up to 95%), a recurrent tumor occurs 2–3 cm from the primary tumor within several months after surgery. New approaches to glioma therapy are being explored to also reduce tumor-cell recovery. Radiation therapy is a critical component of the current combined approach to the treatment of both primary and secondary glioblastomas, increasing the lifespan of patients, which is further improved by targeted therapy. Radiation therapy induces double-stranded DNA breaks in tumor cells, which eventually kills them. At the same time, a fraction of malignant glioma cells are known to be resistant to radiation, and these particular cells prime further tumor growth. The identification of molecular targets of radioresistance mechanisms involving DNA repair can improve the therapeutic efficacy of radiation therapy (2). To date, two DNA repair pathways are involved in tumor-cell recovery, homologous recombination (HR) and nonhomologous end joining (NHEJ) (3). Some researchers claim that HR dominates in radiation-induced DNA damage in glioblastoma cells. This is confirmed by an increased radiosensitivity of glioma cells with inhibited HR, while the inhibition of NHEJ was not as efficient (4, 5). At the same time, many publications indicated the involvement of NHEJ in the repair of radiation-induced DNA damage in glioma cells (6–8). The contribution of these DNA repair pathways to the recovery of glioblastoma cells after radiation therapy remains obscure.

### Homologous recombination (HR)

DNA break repair by HR, typical for normal S and G2 cells, is considered to be more accurate than using nonhomologous end joining (NHEJ) is. One of the main factors of this process is the Rad51 recombinase (38 kDa), which was initially identified in yeast and proved to be an ortholog of the bacterial recombinase A (RecA) (9). The human Rad51 gene was localized to the q arm of chromosome 15 (15.1). Rad51, with a number of factors including Rad52, Rad54, BRCA2, and Rad51 paralogs (XRCC2, XRCC3, Rad51B, Rad51C, and Rad51D), associates with DNA, which allows for error-free break repair. Rad51 displaces replication protein A from long 3’-single-stranded DNA using the mediator proteins Rad52 and Rad55-Rad57 to form a long helical nucleoprotein. Rad51 searches the genome for a homologous target, then Rad51 catalyzes strand invasion forming D-loop, the broken 3’-end fuses with intact homologous template. The broken 3’-end is extended by DNA polymerases using homologous DNA as a template to repair DNA around the break site (10). Rad51 expression levels proved to be higher in tumors than they were in normal cells (11). Rad51 inhibition in human glioma cells substantially increased their radiosensitivity and favored their apoptotic death (2, 5, 12). In addition, the G2 phase was substantially elongated in radioresistant glioblastoma cells exposed to 4 Gγ. And Balbous et al. (2) explored the efficacy of radiation time in contrast to their study using different radiation doses. These studies complement each other in understanding HR significance for cell survival after X-radiation (13).

### Nonhomologous end joining (NHEJ)

A number of different publications claim that NHEJ plays a key role in the repair of radiation-induced double-stranded DNA breaks. In contrast to HR, this repair mechanism can function throughout the cell cycle (14). Normally, NHEJ contributes to the repair of double-stranded DNA breaks in postmitotic cells (15). A variety of proteins are involved in NHEJ, but two play a major role, Ku70 (69 kDa) and Ku80 (83 kDa). Ku70 is encoded by the XRCC6 gene localized on chromosome 22q13.2. The gene has 13 exons and is expressed in nearly all tissue types. Ku80, a subunit of ATP-dependent DNA helicase II, is encoded by XRCC5, localized on chromosome 2q35 (16). These two proteins form a circular heterodimer with the inner diameter corresponding to the DNA double-helix diameter. The Ku70/80 complex has high affinity for double-stranded DNA. After a double-stranded DNA break is formed, the Ku70/80 complex rapidly binds DNA ends, and recruits and activates DNA-PKcs protein kinase to the damage site (17). NHEJ is mediated by at least six major factors, and four, namely, Ku80, Ku70, Ligase IV, and XRCC4, are highly conserved from yeast to mammals. NHEJ is critical for DNA double-strand break (DSB) repair, and thus for genome-stability maintenance. Mice with targeted mutations that inactivate these genes demonstrate phenotypes incapable of such repair (18). All NHEJ-deficient mice suffer from severe combined immunodeficiency. At the same time, deficiencies in NHEJ proteins Ku70 and Ku80 are correlated with the radiosensitivity and high incidence of spontaneous genomic instability. Some researchers claim that NHEJ is more significant for double-stranded DNA repair after the radiation therapy of tumor cells, considering that NHEJ restores double-stranded breaks throughout the cell cycle, while HR is largely limited to the late S/G2 phase (19).

It was proposed that HR and NHEJ are involved in DNA repair in tumor cells after different X-ray doses (20, 21). After low radiation doses (below 40 m Gγ/min), cells use slower but more reliable homologous recombination. As the radiation dose increases, the contribution of HR decreases, and faster but less accurate NHEJ predominates (22).

The goal of this study was to reveal the relationship between high radiation doses and cell proliferation, activation of different repair pathways, and apoptosis.

## Materials and methods

### Glioblastoma cell cultures derived from human tumors

Primary cultures were obtained from human postoperative glioblastoma complying with all formal requirements of the Russian Federation. This study was approved by the Ethics Committee of Burdenko Neurosurgical Institute, Russian Academy of Medical Sciences (№_12/2020). All subjects gave written informed consent in accordance with the Declaration of Helsinki.

Human glioma samples were transported to the laboratory within 1 h after surgery in DMEM/F12. Samples were transferred to a Petri dish, dissected, released from vessels, washed with Versene, and incubated with 0.25% trypsin (PanEko, Russia) at 37°C with agitation for 40 min. After dissociation for three times, the supernatant was centrifuged at 1000 rpm for 5 min. Filtered and centrifuged cells were cultured in DMEM/F12 (Gibco, Germany) with 10% FBS (HyClone, USA) and plated in flasks. One day later, unattached cells were removed, and fresh medium was added. Subsequently, the medium was replaced once in 3 days. L-glutamine (PanEko, Russia) was added to the DMEM/F12 medium at 300 mg/l. Cell cultures were incubated at 37°C with 5% CO2. Cultures were maintained for up to 20 passages, and a fraction of cells were cryopreserved at each stage.

### Immunocytochemistry

Neurospheres of glioblastoma cells were washed twice in PBS (pH 7.3). Staining was performed using the following primary antibodies: rabbit polyclonal Nestin antibody (dilution 1:200, Chemicon, USA), mouse monoclonal Vimetntin antibody (dilution 1:100, Sigma-Aldrich, USA). The primary antibodies were dissolved in PBS with 0.3% Triton X100 (Sigma-Aldrich, USA), plus 2% donkey serum (Jackson Immunoresearch, UK), and were incubated for 2 h at room temperature. A solution with 1% FBS and 2% donkey serum was used as a negative control. After washing three times for 5 min with PBS (pH 7.3), cells were incubated for 1 h with the following secondary antibodies: donkey anti-rabbit antibodies conjugated with Alexa Fluor 594 (dilution 1:100, #711-545-152, Jackson Immunoresearch, UK), donkey anti-mouse antibodies conjugated with DyLight-488 (dilution 1:100, #705-585-147, Jackson Immunoresearch, UK). Then, neurospheres were washed with PBS (pH 7.3) and stained with bisbenzimide (Sigma- Aldrich, USA) for 5 minutes at room temperature. The cells were washed with PBS (pH 7.3), covered with glycerin, and analyzed by fluorescent microscopy. An Olympus IX81 (Olympus corp., Japan) microscope was used for visualization with a computer-controlled motorized stage (Märzhäuser, Wetzlar) and an Olympus DP72 digital camera (Olympus, Münster, Germany).

### X-radiation

For each beam parameter, a 96-well plate, two flasks, and a dish were irradiated using linear accelerator TrueBeam STx (Varian, USA), commonly used in medical practice and receiving regular technical maintenance. Phantoms were exposed to a single vertical bremsstrahlung radiation with a rated energy of 6 MeV and a power of 600 dose rate/min. Field size of 32 × 32 cm provided for uniform radiation of the entire culture. The SSD was 98 cm. Radiation doses varied from 1 to 250 Gγ. Exposure duration depended on the dose and varied from 10 s (1 Gγ) to 43 min (250 Gγ). Control cells were not exposed to radiation but kept under the same conditions, including transportation.

### MTT

Colorimetric MTT test was used to estimate cell viability; 96-well plates with 3 × 103 cells per well were incubated at 37°C for 24 h. After X-ray irradiation, plates were incubated for five days. Then, 20 μl of 5 mg/ml 3-(4,5-dimethylthiazol-2-yl)-2,5-diphenyltetrazolium bromide (Sigma-Aldrich, USA) was added, and plates were incubated at 37°C for 2 h. After removal of the medium, 60 μl/well of DMSO (PanEko, Russia) was added, stirred for 15 min, and A 540 nm was measured with a Tecan plate reader (Switzerland). The background with MTT for nonirradiated cells was subtracted, and the mean ± SEM of five replications was used.

### Reverse transcription and qPCR assay

Expression levels of XRCC5, XRCC6, and RAD51 in primary glioma cultures were quantified by real-time PCR. Total RNA was isolated from glioma cells using RNAzol solution (MRC, USA) according to the manufacturer’s protocol. cDNA was synthesized from isolated total RNA using the MMLV RT kit (Evrogen, Russia) according to the manufacturer’s protocol. cDNA was amplified on a СFX96 Real-Time PCR Detection System (Bio-Rad, USA) using a kit with the SYBR Green I dye (Syntol, Russia). PCR was carried out in a volume of 25 µl containing 2.5 µl dNTPs (2.5 mM each), 1xPCR buffer B, primers (0.25 mM each), and 1 U of Taq DNA polymerase. PCR conditions were as follows (41 cycles): (1) denaturation at 94°C for 20 s, (2) annealing 61°C for 20 s, and (3) 30 s extension at 72°C. Fluorescence was recorded after Step 3. Melting curves were generated after amplification to confirm the homogeneity of the PCR product. RT-PCR data were analyzed using the software supplied with the СFX96 System (Bio-Rad, USA). Primers were selected from RTPrimerDB and using Primer-Blast (NCBI). The efficiency of the selected primers (specificity, dimerization, etc.) was evaluated by the agarose gel electrophoresis of PCR products. Housekeeping gene HPRT was used as the control.

The following primers were used:

Rad51_F 5’-CAGCTGGGAACTGCAACTCA-3’

Rad51_R 5’-ACCGTGAAATGGGTTGTGGG-3’

XRCC5_F 5’-TGACTTCCTGGATGCACTAATCG-3’

XRCC5_R 5’-CCTAAGCGAAAGGGGCCAT-3’

XRCC6_F 5’-GTTGATGCCTCCAAGGCTATG-3’

XRCC6_R 5’-CCCCTTAAACTGGTCAAGCTCTA-3’

HPRT_F 5’-TGAGGATTTGGAAAGGGTGT-3’

HPRT_R 5’-GAGCACACAGAGGGCTACAA-3’

### Western blotting

Studied cells were harvested by 0.25% trypsin, washed twice with PBS, and lysed in buffer containing 8 M urea, 1% (v/v) Triton X-100, and 50 mM DTT. After centrifugation at 21,000g for 20 min, the supernatant was transferred to a new tube and used as a stock solution. Aliquots of the stock solution were supplemented with 5 volumes of Laemmli buffer and analyzed by electrophoresis in 10% acrylamide gel. The transfer to a nitro-cellulose membrane (Bio-Rad, USA) was performed using a Mini Trans-Blot System (Bio-Rad, USA) in a buffer containing 25 mM Tris, 192 mM glycine, and 20% (v/v) ethanol. The membrane was incubated in 1% Blocking reagent (Roche #11 096 176 001, Germany) for 50–60 min. Then, the membrane was incubated overnight with Cell Signaling antibodies: aRad51 (d4810), 1/10000; aKu70 (d35), 1/2000; aKu80, 1/8000; and beta-tubulin (9F3), 1/2000. The membrane was washed thrice with TBS-T and exposed to the secondary horseradish peroxidase-conjugated antibodies (aRb*HRP Santa Cruz sc-2030; 1/10000) for 1 h. The signal was detected using the ECL system (Amersham). Western blot data were analyzed by ImageJ according to the software instructions (https://imagej.nih.gov/ij/download.html).

### Flow cytofluorometry

Cell death (apoptosis and necrosis) was evaluated using the Annexin V-FITC kit (Biolegend, USA) in accordance with the manufacturer’s instructions. Apoptotic events were detected on a BD Accuri C6 flow cytometer (BD, USA). Data were processed using CFlow software (BD, USA). After applying the standard fluorescence-compensation technique, the percentage populations of Annexin V +/PI + cells in the scatter plot with two parameters were used for statistical analysis (30,000 events were collected in each probe designated as “target cells” in the FSC-SSC chart).

### Statistical analysis

Results were expressed as mean ± standard error of the mean (SEM). The statistical evaluation was performed by Mann-Whitney test. Each value is the mean of ≥3 independent experiments ± SEM.

## Results

Experiments were carried out on two cell cultures derived from supratentorial glioblastoma patients.

**Figure 1:**
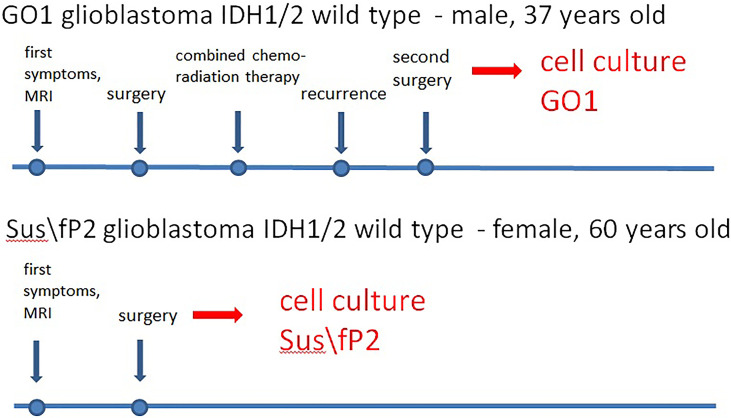
Characteristics of glioblastoma cell cultures.

**Figure 2:**
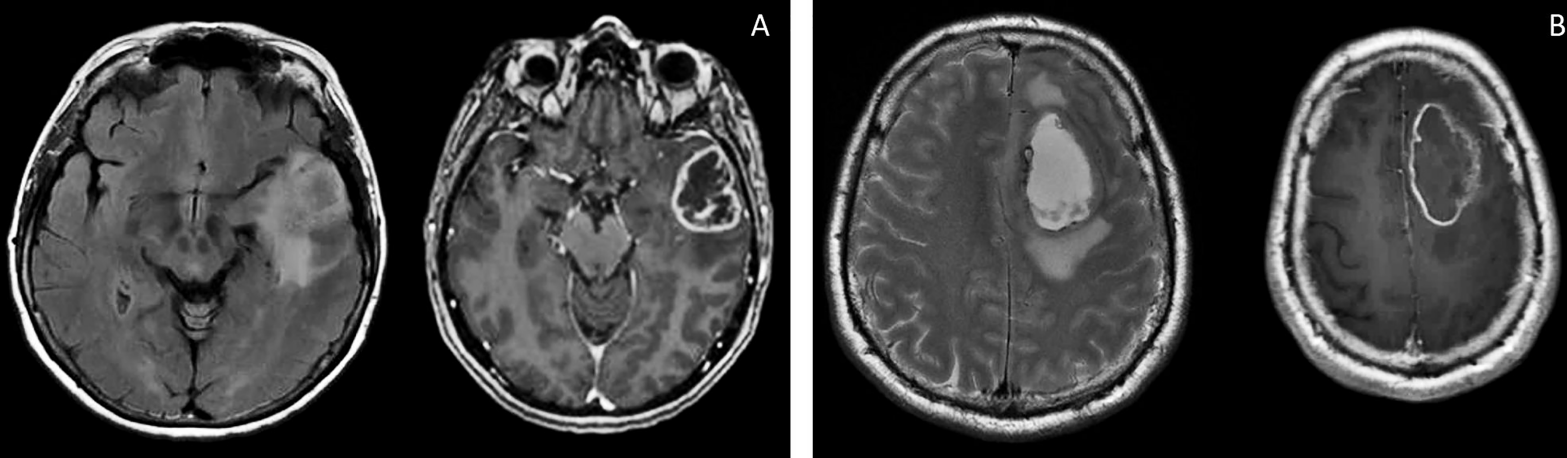
Brain MRI, T2 FLAIR, and T1 images of patients S **(A)** and G **(B)**. There are the same typical heterogenic changes of the signal with a ring-like contrast enhancement and a signal typical for edema around the tumors.

Patient S, female, 60 years old, had a short medical history. Headaches and amnestic disorders appeared and progressed within two weeks, and a mass lesion was found in the left temporal lobe with a circular magnetic resonance tomography (MRT) pattern (**Figure 2**), after which subtotal removal of the tumor was performed. Sanger sequencing indicated IDH1/2 wild type. A continuous cell culture Sus\fP2 for up to 20 passages was derived from the tumor. The patient received chemoradiation therapy with a total radiation dose of 60 Gγ and subsequent administration of temozolomide, which was canceled after two courses due to hematological toxicity. Eight months after chemoradiation therapy, clinicoradiological analysis demonstrated progressive tumor growth, and chemotherapy and targeted second-line treatment (bevacizumab) were administered. However, the patient died of tumor progression at 19 months after diagnosis (**Figure 1**).

Patient G, male, 37 years old, had a sudden epileptic seizure. MRT (**Figure 2**) demonstrated a typical ring-shaped gadolinium enhancement in the left frontal lobe with typical ring gadolinium enhancement, after which subtotal removal of an IDH1/2 wild-type glioblastoma was performed. The patient received chemoradiation therapy with a total radiation dose of 66 Gγ and subsequent administration of temozolomide. Twenty-four months after the first surgery, the patient was reoperated due to a locally recurrent tumor. Cell culture GO1 for up to 20 passages was derived from the tumor. Chemoradiation treatment with a total radiation dose of 66 Gγ, combined with temozolomide, bevacizumab, and irinotecan was performed. The patient died of tumor progression at 41 months after the first diagnosis or at 17 months after the second operation and cell-culture preparation (**Figure 1**).

Thus, the Sus\fP2 cell culture was derived from a glioblastoma not exposed to radiation, while the GO1 culture was derived from a recurrent glioblastoma after X-ray therapy.

**Figure 3:**
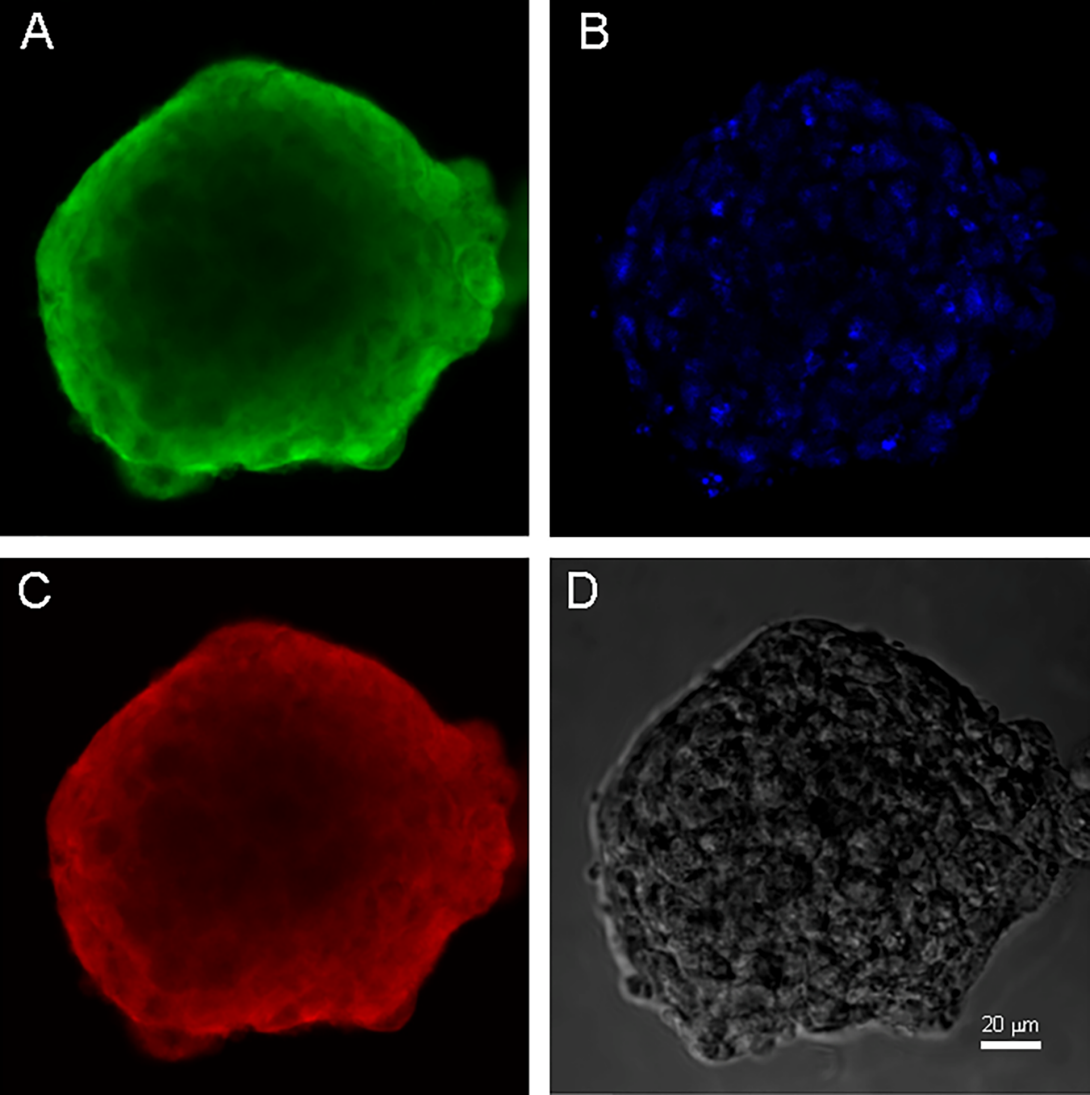
Neurospheres of glioblastoma cell culture of GO1. **(A)** antibody staining for Vimentin. **(B)** staining for bisbenzimid, **(C)** antibody staining for Nestin, **(D)** neurospheres in phase contrast. Scale bar is 20 μm.

**Figure 4:**
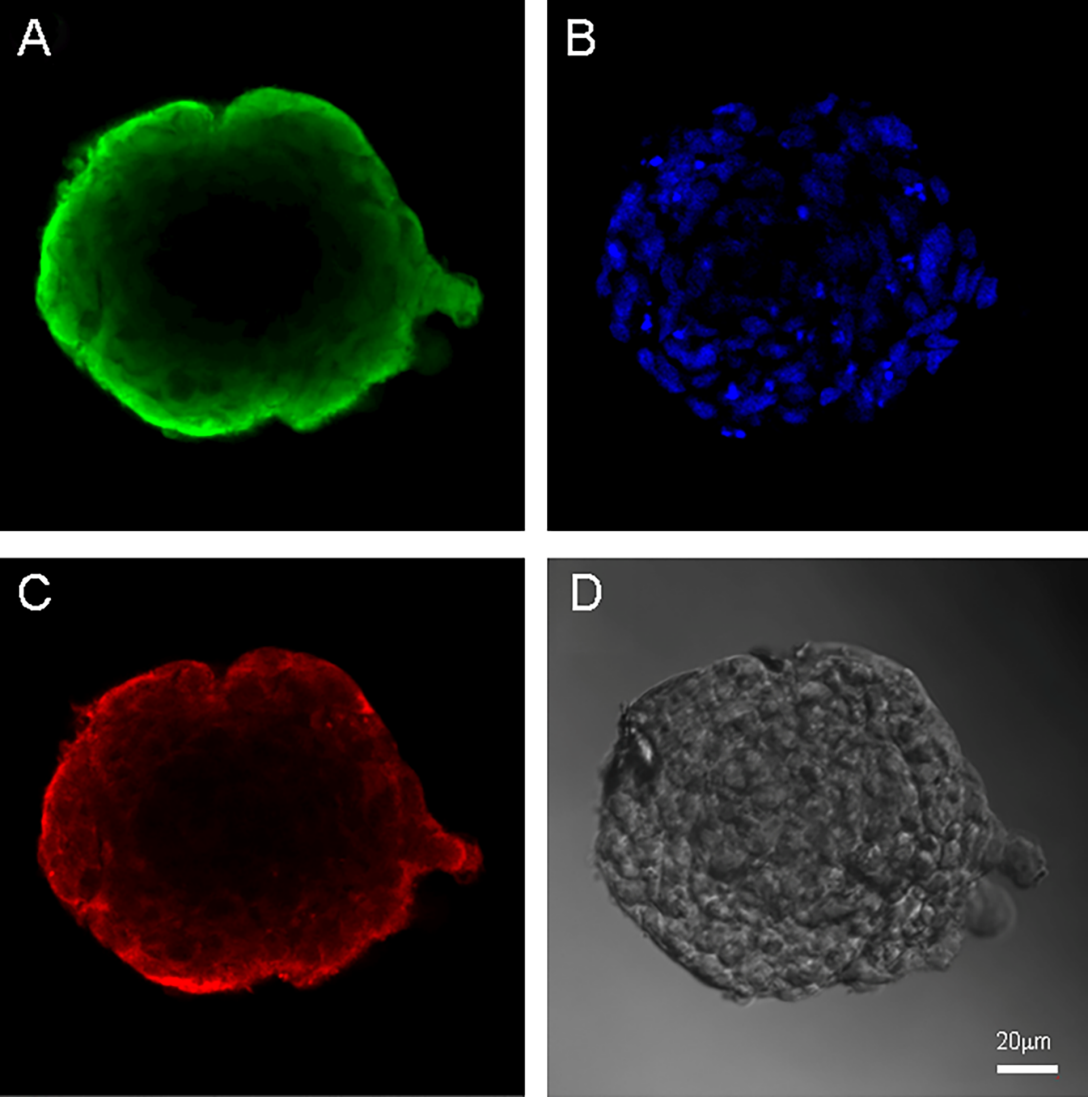
Neurospheres of glioblastoma cell culture of Sus\fP2. **(A)** antibody staining for Vimentin. **(B)** staining for bisbenzimid, **(C)** antibody staining for Nestin, **(D)** neurospheres in phase contrast. Scale bar is 20 μm.

Both cell cultures were able to form neurospheres with positive staininf for Nestin and Vimentin (**Figures 3**, **4**). Cell cultures were characterized with an expression of the critical genes of human glioblastomas (**Supplementary Figure 1**).

### Effect of radiation on cell proliferation

The obtained cell cultures were maintained for up to 10–20 passages. These cultures were heterogeneous, which distinguishes them from most experimental cell cultures. This brings our experiment closer to *in vivo* processes going into actual tumors exposed to radiation.

The goal of this study was to investigate the effect of single exposure to a wide range of X-ray doses (from 0 to 250 Gγ) of glioblastoma cultures derived from excised primary tumors. Changes in DNA repair capacity were studied under these conditions. The second goal was to investigate the susceptibility of glioma cells to fractionated radiation (two fractions). A special phantom was designed and fabricated from water-equivalent material with a size of 30 × 30 × 3 cm composed of two 1.5 cm plates, with the required number of pockets coinciding with the cell-culture vessel. The Hounsfield value of the phantom material was 110. Uneven exposure in the build-up region was excluded by two solid water slabs with a total thickness of 2 cm on the top and a 5 cm solid water slab on the bottom. Exposure was carried out using a clinical unit with a high dose rate and up-to-date beam characteristics for glioblastoma radiation therapy. Exposure planning and performance approximated clinical radiation procedures (23).

**Figure 5:**
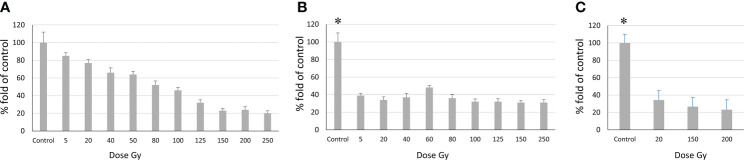
Relationship between the number of proliferating cells and radiation dose according to the MTT assay. **(A)** Sus\fP2 cells; **(B)** GO1 cells, **(C)** Sus\fP2 cells exposed to radiation below 5 Gγ. Each value is the mean of ≥3 independent experiments ± Standard Error of the Mean (SEM). Control values on **(B, C)** (denoted with *) significantly exceeded the experimental ones at any radiation doses with p<0.01 according to the Mann-Whitney test.

At the first stage, single exposure was used to analyze the effect of radiation on cell proliferation in the culture and on the DNA repair capacity of cells, using the MTT assay. Both the major HR pathway of DNA repair involving Rad51 and the NHEJ pathway involving the Ku proteins were studied. The population of proliferating cells decreased with the dose of single exposure (**Figure 5**). GO1 and Sus\fP2 cells not exposed to radiation served as control.

Obtained data demonstrated a sharp decrease in the proliferative activity of Sus\fP2 cells in the dose range from 1 to 40 Gγ relative to GO1 cells. As the dose was increased to 250 Gγ, the proportion of viable cells remained stable at the level of about 30%. This indicated that a pool of cells highly resistant to radiation was stably maintained.

In contrast to Sus\fP2, the proliferative activity of the GO1 culture decreased more smoothly with the radiation dose up to 100 Gy. At a dose of 150–250 Gγ, the proportion of metabolically active cells was about 20%.

The revealed sharp decrease in Sus\fP2 proliferative activity as opposed to G01 prompted us to further test the exposure of Sus\fP2 cells to radiation below 5 Gγ in smaller steps (**Figure 5C**).

**Figure 6:**
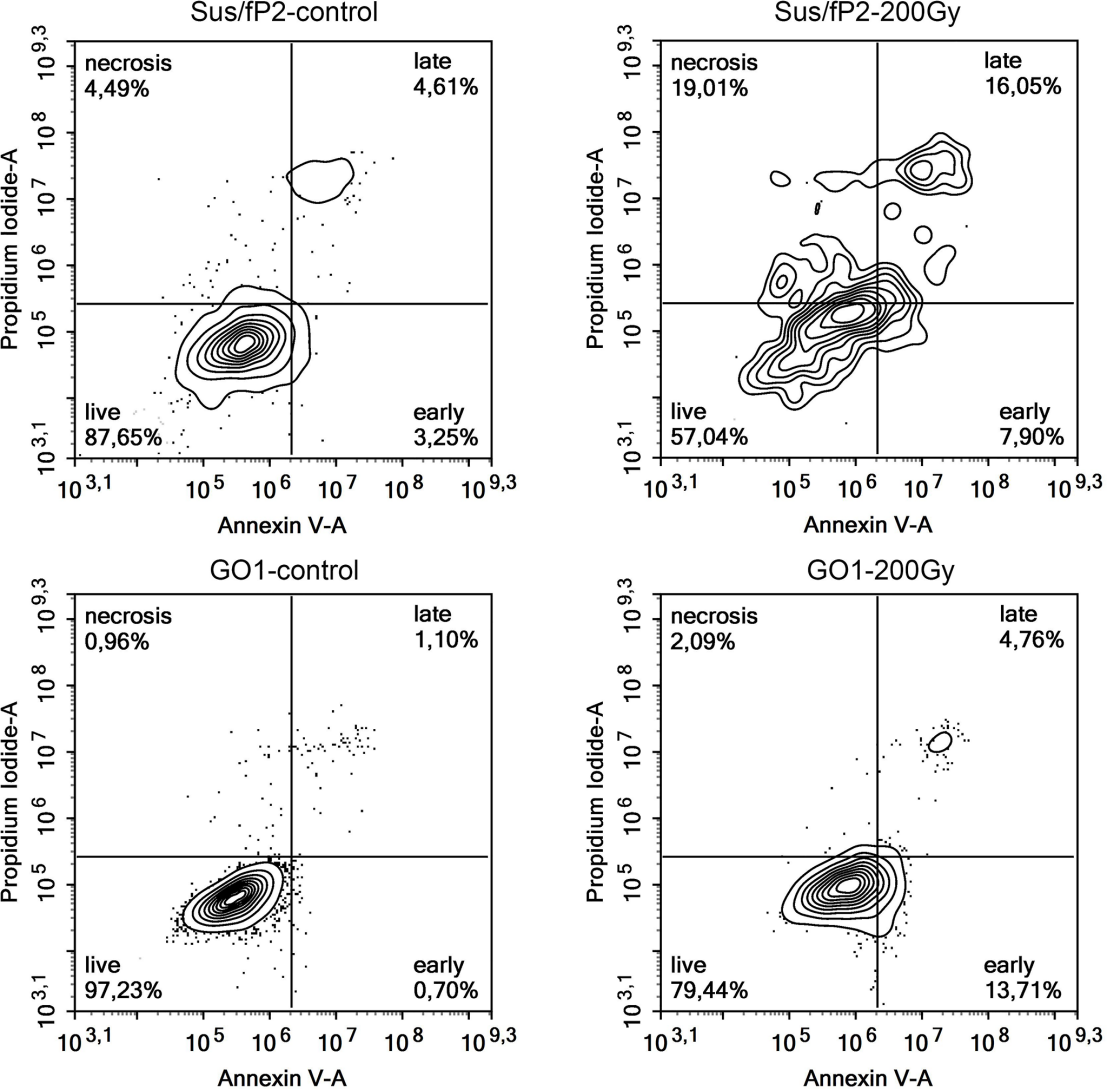
Analysis of apoptosis and necrosis in Sus\fP2 and GO1 cell cultures exposed to 200 Gγ.

This supplementary experiment confirmed the decrease in Sus\fP2 proliferative activity as the radiation dose increased from 0 to 5 Gγ. The significance of apoptosis and necrosis in cell death after radiation was evaluated using a high dose of 200 Gγ. After 7 days, cell death was evaluated using flow cytofluorometry. The necrotic and apoptotic indices of cultures substantially differed (**Figure 6**).

The proportion of Sus\fP2 cells that died *via* apoptosis was insignificantly higher than that *via* necrosis. In the GO1 culture, the proportion of apoptotic cells was much higher than that of necrotic cells (which were close to 0%). Both cultures included cell populations not responding to radiation by self-destruction.

The relationship between DNA repair and radiation dose was studied. In the case of HR typical for DNA repair in both irradiated and nonirradiated tumor cells, the expression level of the primary gene involved, RAD51, and the level of its protein product were studied in irradiated and nonirradiated tumor cells. In the case of NHEJ, the expression levels of the major genes involved, XRCC6 and XRCC5, and the levels of their products, Ku70 and Ku80 were analyzed.

### Evaluation of DNA repair by homologous recombination

**Figure 7:**
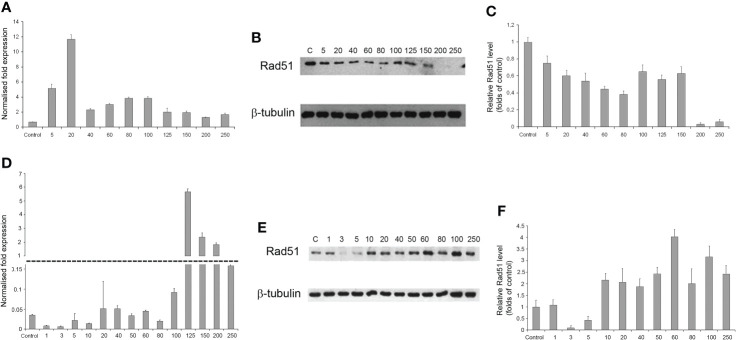
The dependence of RAD51 mRNA and protein levels on radiation dose in GO1 **(A–C)** and Sus\fP2 **(D–F)** glioblastoma cell cultures. **(A, D)** mRNA expression; **(B, E)** Western blotting; **(C, F)** Quantification RAD51 protein levels by ImageJ.

As the radiation dose increased to 250 Gγ, RAD51 transcription in GO1 cells varied insignificantly (except a significant burst at 20 Gγ; **Figure 7A**). Analysis of RAD51 transcription revealed no dose-dependent effect; however, its level was higher than that in the control in all samples. The level of RAD51 protein showed a dose-dependent decrease (**Figures 7B, C**). The increased gene activity can be attributed to alternative splicing producing proteins not recognized by antibodies against RAD51 in Western blotting. The decreased level of the RAD51 protein can indicate that high radiation doses decrease the rate of HR-mediated DNA repair. The second studied culture, Sus\fP2, demonstrated significant growth in RAD51 transcription at doses exceeding 100 Gγ. RAD51 proved most sensitive to doses of 3 and 5 Gγ, as confirmed by both RT-PCR and Western blotting. The same doses substantially de-creased proliferation in this culture (**Figure 5A**). Conversely, a further increase in radiation promotes an increase in RAD51 expression.

Thus, the patterns of RAD51 expression were significantly different in tumor-cell cultures pre-exposed (GO1) or not pre-exposed (Sus\fP2) to radiation (**Figures 7D–F**).

### Evaluation of DNA repair by nonhomologous end joining

**Figure 8:**
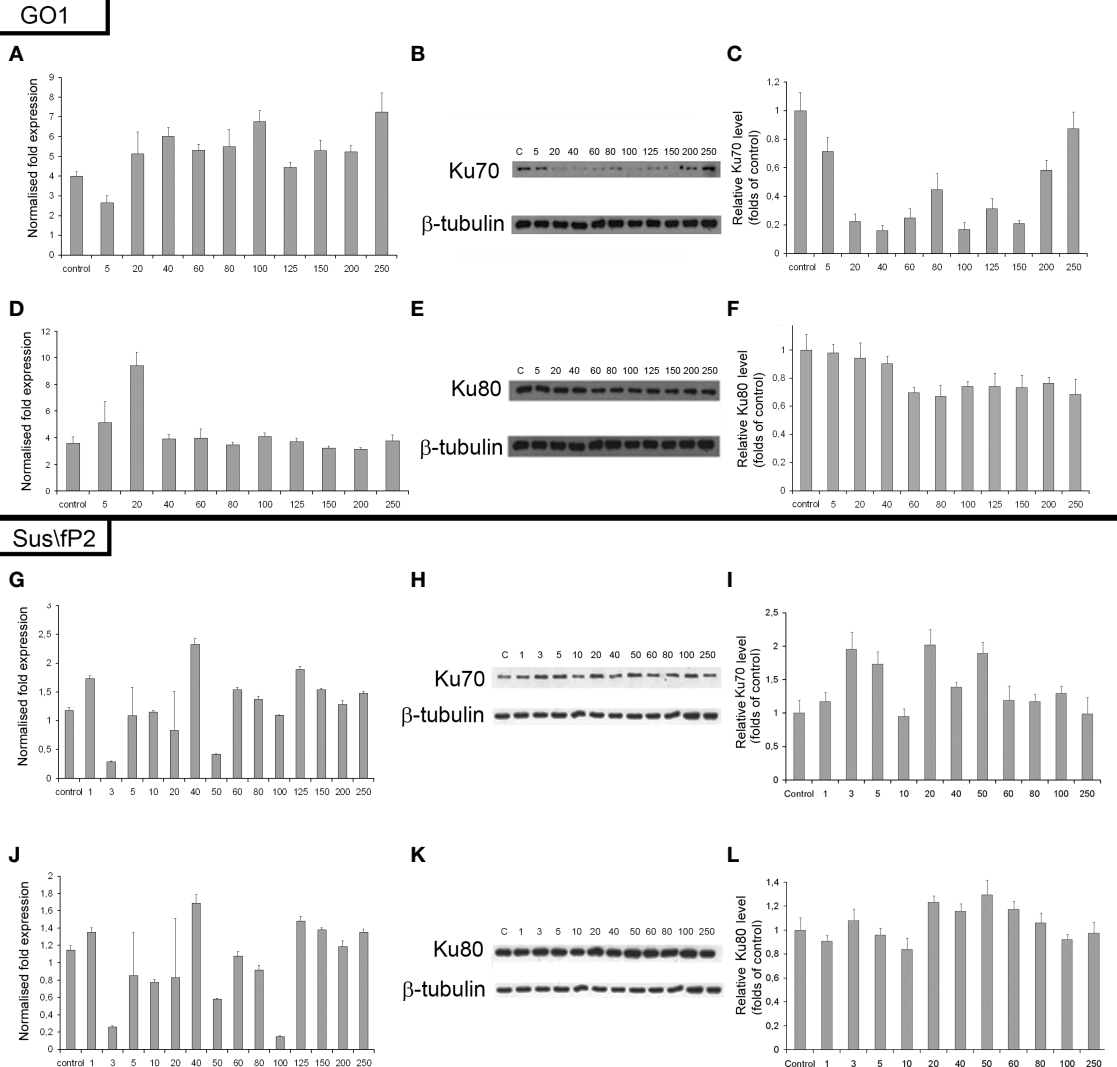
Expression of XRCC6 gene **(A)** and its product Ku70 **(B, C)** as a function of radiation dose in glioblastoma GO1 cell culture. Expression of XRCC5 gene **(D)** and its product Ku80 **(E, F)** as a function of radiation dose in GO1 cell culture. Expression of XRCC6 gene **(G)** and its product Ku70 **(H, I)** as a function of a single radiation dose from 5 to 250 Gγ in glioblastoma Sus\fP2 cell culture. Expression of XRCC5 gene **(J)** and its product Ku80 **(K, L)** as a function of a single radiation dose from 5 to 250 Gγ in Sus/fP2 cells.

The expression of genes and proteins is involved in DNA repair *via* NHEJ after exposure to different radiation doses. Ku70 and Ku80 are critical factors in this pathway; accordingly, the expression of these proteins and their genes, XRCC6 and XRCC5, was investigated (**Figure 8**). The expression of Ku80 in GO1 proved insensitive to radiation dose (Figures 8D–F), as indicated by insignificant variations in the levels of both XRCC5 mRNA and the Ku80 protein.

Exposure to radiation doses from 1 to 40 Gγ decreased the protein expression of Ku70 (**Figures 8A–C**). Doses from 40 to 150 Gγ induced minor variations on the protein level; however, this was lower than that in the control. Doses from 200 to 250 Gγ increased protein synthesis. Analysis of XRCC6 mRNA demonstrated no significant variations on its level. RT-PCR detects the expression of all XRCC6 transcripts; thus, the steady mRNA level could be attributed to alternative splicing decreasing the proportion of mRNA species translated into Ku70.

NHEJ analysis in a tumor-cell culture not pre-exposed to radiation (Sus\fP2) demonstrated no significant changes in the expression of proteins Ku70 and Ku80 (**Figures 8G–L**).

Considering our previous data indicating that decreased RAD51 expression is correlated with decreased proliferation of Sus\fP2 cells after exposure to up to 10 Gγ, the role of NHEJ in tumor-cell survival seems insignificant.

## Discussion

Radiation therapy is a critical component of the current combined approach to the treatment of glioblastomas. An important impact of radiation on living cells is DNA breaks. Significant DNA damage by double-stranded breaks can be expected to induce apoptosis and cell death. However, DNA repair in damaged cells allows for them to survive. DNA repair should be studied in the recovery of glioblastoma cells to reveal pathways critical for their survival and the dose dependency of their effect. It is common knowledge that two DNA repair pathways are involved in tumor-cell recovery after radiation therapy. The homologous recombination (HR) pathway is considered critical for the normal development and functioning of the body, and for the repair of damaged DNA in tumor cells after radiation or chemotherapy. One of the main proteins involved in HR is RAD51, and we studied its mRNA and protein expression here. Another mechanism of DNA break repair that is considered more typical for tumor cells (8) is nonhomologous end joining (NHEJ). The major components of NHEJ are the Ku70 and Ku80 proteins encoded by the XRCC5 and XRCC6 genes, respectively. Ku70 and Ku80 form a circular heterodimer with a central barrel that can cradle the DNA helix (24). Holoenzyme Ku70/80 is the heterodimeric component of DNA-dependent protein kinase (DNA-PK). A number of studies not dealing with cancer-cell irradiation showed that inactivation of DNA-PK yielded a higher level of radioresistance even after 24–72 h of repair. A number of factors were identified that interfere with DNA-PK interactions and increase radiosensitization (25).

**Figure 9:**
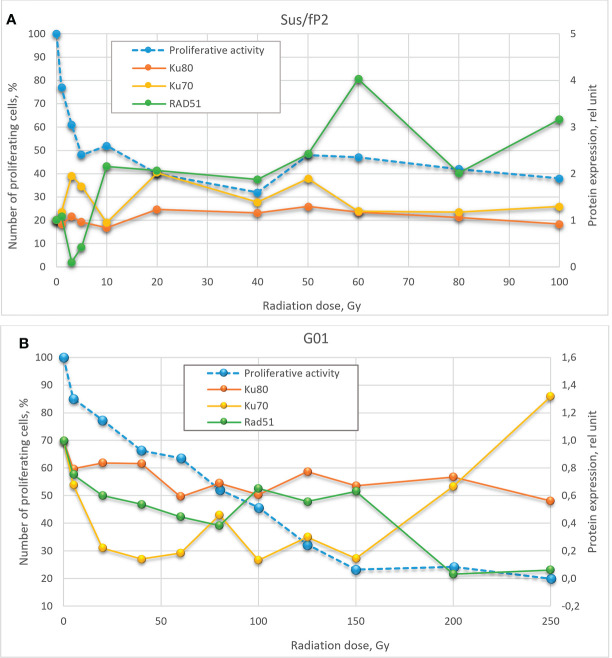
Dose-effect relationship between Sus\fP2 **(A)** and GO1 **(B)** proliferative activity and expression of RAD51, Ku70, and Ku80 proteins.

Here, we studied changes in the expression of these proteins and genes as a function of radiation doses in the range from 1 to 250 Gγ. We found no publications on DNA repair in glioblastoma cells exposed to a similar dose range. Experiments were carried out on two cell cultures derived from human patients diagnosed with glioblastoma. These are not cell lines, i.e., not uniform. We tried to preserve their heterogeneity, and they were not maintained for more than 20 passages. One of these cultures (GO1) was derived from a recurrent tumor that was exposed to radiation, unlike Sus\fP2. Analysis of their proliferative activity after exposure to 1 to 250 Gγ demonstrated higher sensitivity of Sus\fP2 to radiation; the proliferative capacity of its cells decreased much faster to reach the bottom at 40 Gγ. Significantly, this notable proliferative decline was accompanied by the decreased expression of HR protein factor RAD51 (**Figure 9A**). GO1 proliferative activity decreased much slower and reached the bottom at 150 Gγ, which could indicate the higher radioresistance of cells acquired after the preceding exposure. Similar dose dependence was observed for the expression of the RAD51 protein.

While many researchers (19) indicated an insignificant role of HR in DNA repair in tumor cells, we observed a correlation between the proliferative activity of cells and the expression level of the main factor of HR-mediated DNA repair, RAD51.

Analysis of proliferative activity in two studied cell cultures in the context of the expression of proteins Ku70 and Ku80, markers of NHEJ involvement in tumor-cell survival after radiation, demonstrated no increase in Ku80 protein level with a radiation dose in both cultures. Considering that the Ku70/Ku80 heterodimer is involved in NHEJ, and Ku80 is always excessive, it cannot be used as a marker in this case. No clear relationship between the proliferative activity of Sus\fP2 and GO1 cells, and Ku70 protein expression was revealed.

Comparison of the dose–effect curves for the expression of proteins critical for HR- and NHEJ-mediated DNA repair and proliferative activity (MTT) further confirmed our observations.

Obtained data after radiation of a cell culture not pre-exposed to X-ray therapy corroborate the proposal by Sasaki et al. (20) that cell proliferation varies with the expression of the RAD51 protein. Hence, it is critical for DNA repair at doses below 40 Gγ, which is apparent in **Figure 9** for doses below 10 Gγ. As the dose increases above 40 Gγ, the proliferative activity of cells varies with the protein levels of NHEJ factors Ku70 and Ku80, but no such relationship was observed between proliferative activity and RAD51 expression, which suggests that NHEJ is the main DNA repair pathway at high radiation doses. Mladenov made the same suggestion when using lower doses (26). However, they found a switch of HR-mediated DNA repair to NHEJ, which could be explained with using linear low-diversity cancer cells A549, HA-AsiSI-ER-U2OS, HCT116, and U2OS (γ27).

Different patterns of HR and NHEJ involvement in DNA repair were observed in the tumor pre-exposed to radiation. GO1 cell proliferation tended to decrease with radiation dose in a way similar to that for the expression of the RAD51 protein. No such pattern was observed for the expression of the Ku70 and Ku80 proteins (**Figure 8B**). This means that HR plays the leading role in DNA repair in this case, and this pattern is no longer observed only after high radiation doses (above 100 Gγ). GO1 cells demonstrate high radiation resistance, and their proliferative activity decreases slowly with radiation doses.

Thus, we demonstrated an important role of RAD51, and hence HR, in DNA repair in cells after low-dose therapy radiation (for Sus\fP2, up to 40 Gγ; for GO1, up to 80 Gγ). RAD51 is considered to mediate only the slow repair of double-stranded DNA breaks and is critical for DNA replication (27). However, our data confirmed the proposal of Short et al. (28) that the level of RAD51 protein is relevant for the radioresistance of glioblastoma cells, which could be modulated by decreasing the cellular level of the RAD51 protein (12). Zhong et al. (29) (came to similar conclusions concerning nonsmall cell lung cancer. The latter publication also demonstrated that RAD51 knockdown enhances the radiation-induced degradation of tumor cells and induces their apoptosis.

The significance of RAD51, and hence HR, decreases with high doses of radiation, and cell proliferation starts to depend on Ku70 expression, which indicates the increasing importance of the NHEJ pathway. The radiation dose switching from HR to NHEJ substantially differs in tumor cells pretreated and not pretreated with radiation.

We have applied glioblastoma-derived cell cultures to demonstrate, for the first time, changes of sensitivity of tumor-cell to radiation dose. We have shown that different glioblastomas respond differently to radiation therapy, and this could be due to their peculiarities. For example, a sensitivity of tumor cells to radiation could change if the patient has previously received radiation therapy. Other characteristics of the tumor could also modulate either resistance or sensitivity to radiation therapy. Nevertheless, it becomes possible to choose a more effective dose of radiation for a patient’s tumor studying the corresponding individual cell cultures. Our data could justify an individual approach to radiation therapy for a patient.

The significance of a patient-specific approach was confirmed by several publications, e.g., a recent one describing the radiation effect on apoptosis and necrosis in prostate cancer cell lines (30), which demonstrated that low and high radiation doses efficiently induce apoptosis and necrosis, respectively. The exposure of Sus\fP2 to a high dose (200 Gγ) corroborates this conclusion: most cells underwent necrosis. A similar experiment on a cell culture derived from a tumor pre-exposed to radiation demonstrated a different pattern: most dying cells underwent apoptosis. Hence, cell-death processes can vary with treatment conditions and individual neoplasm properties.

Our second observation is that tumor cells pre-exposed to radiation can be more radiation-resistant, which is mediated by HR and hence RAD51. This agrees with the proposal of Short et al. (28) that RAD51 is more significant for DNA repair in tumor cells exposed to radiation than Ku70/Ku80 is. We found the importance of HR repair at irradiation doses from 1 to 40 Gγ for a glioblastoma cell culture without preliminary irradiation and from 1 to 100 Gγ for a culture with preliminary irradiation.

Our study agrees with the viewpoint that the control of HR processes in human glioma cells is more efficient than the inhibition of the main alternative pathway of DNA repair, NHEJ (4, 5, 31).

## Conclusion

Radiation therapy is a critical component of the current approach to glioblastoma (GBM) treatment. However, some GBM cells recover after this exposure and initiate tumor growth. The substantial point is to evaluate a contribution of DNA repair pathways, homologous recombination (HR) and nonhomologous end joining (NHEJ) to a restoration of GBM tumor cells. There is a need to understand to what degree GBM cells change in their sensitivity to radiation during repeated radiation exposure. The point is to conduct this kind of studies not on linear GBM cells, but on primary cell cultures from patient tumors, paying attention to the small number of passages. We used GBM-derived cell cultures to demonstrate, for the first time, changes in tumor-cell sensitivity to radiation doses in the range of 1–250 Gγ. We also suggested an important role of RAD51, and hence HR, in DNA repair in cells after radiation therapy (up to 80 Gγ). The significance of RAD51 and hence HR, decreases with high doses of radiation, and cell proliferation begins to depend on Ku70 expression, which indicates the increasing importance of the NHEJ pathway. Radiation dose switching a mechanism (reparative process) from HR to NHEJ substantially differs for two cell cultures, possibly due to a status of being either pre-treated with radiation or not pre-treated one.

## Data availability statement

The original contributions presented in the study are included in the article/**Supplementary Material**. Further inquiries can be directed to the corresponding author.

## Ethics statement

The study involving human participants (cell cultures) was conducted according to the guidelines of the Declaration of Helsinki and was approved by the Local Ethics Committee of Burdenko Neurosurgery Center (protocol code №12/2020, 15.12.2020) and with the 1964 Helsinki declaration and its later amendments or comparable ethical standards. The patients/participants provided their written informed consent to participate in this study.

## Author contributions

GP, AB, and AG contributed to the whole conception and design of this project. AP worked as the neurosurgeon to collect cases and analyze the clinical data. DG selected the tissue of human glioblastoma for preparing cell cultures from the Biobank that he directs. ES was responsible for the preparation of glioblastoma cell cultures derived from human tumors for research and MTT. AB, AN, and NA performed X-radiation; DS: reverse transcription and qPCR assay; DP and AR: Western blotting; AR and ES: flow cytofluorometry. GP completed the manuscript and figures and formed the concept of the article; AR helped in figure design. All authors were involved in the writing of the manuscript at the draft and revision stages and have read and approved the final version. All authors have read and agreed to the published version of the manuscript.

## Funding

This research was funded by the Ministry of Science and Higher Education of the Russian Federation, grant number 075-15-2020-809 (13.1902.21.0030).

## Acknowledgments

We are grateful to Marina Ryzhova (N.N. Burdenko NMRCN) for evaluating IDH mutations in cell cultures.

## Conflict of interest

The authors declare that the research was conducted in the absence of any commercial or financial relationships that could be construed as a potential conflict of interest.

## Publisher’s note

All claims expressed in this article are solely those of the authors and do not necessarily represent those of their affiliated organizations, or those of the publisher, the editors and the reviewers. Any product that may be evaluated in this article, or claim that may be made by its manufacturer, is not guaranteed or endorsed by the publisher.
