# Supplementary material for: Reparative properties of human glioblastoma cells after single exposure to a wide range of X-ray doses
Source: Front Oncol. 2022 Aug 4;12:912741. doi: 10.3389/fonc.2022.912741 (PMC9386365; doi:10.3389/fonc.2022.912741)
Supplement: Supplementary file 1 [file DataSheet_1.pdf]

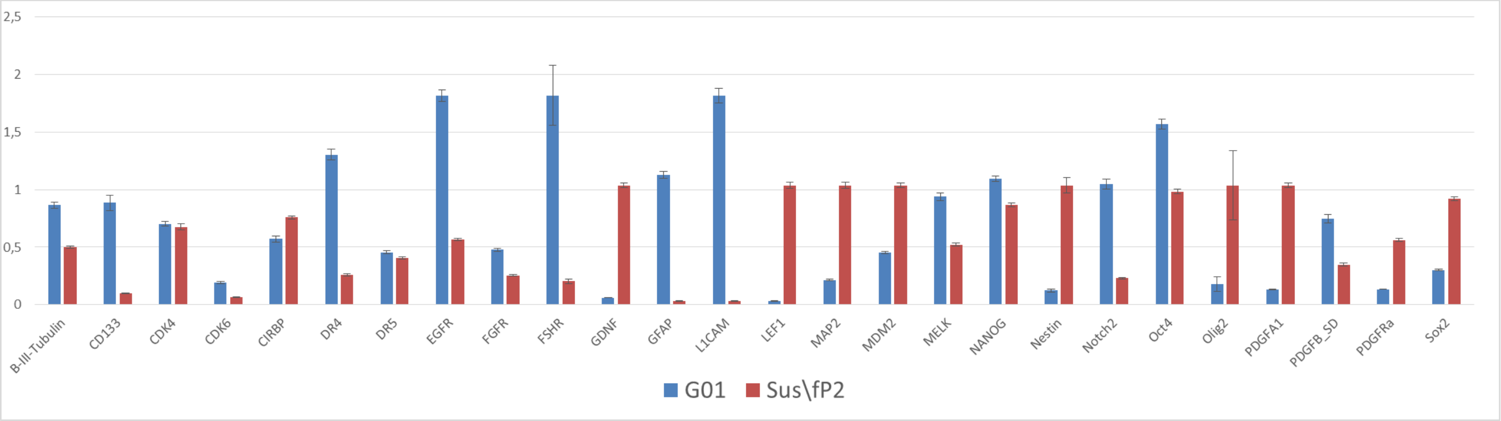

Supplementary Figure 1. Expression of some oncogenes and tumor stem cell genes in glioblastoma cell cultures GO1 and Sus\fP2.
